# Use of an innovation center to foster high‐value COVID‐19 care at an academic healthcare system

**DOI:** 10.1002/jhm.12824

**Published:** 2022-05-08

**Authors:** Melissa E. Shumacher, Sharon Markman, Kayla Scales, Laura Fritsche, Kimisha Cassidy, Jane L. Holl, Craig A. Umscheid

**Affiliations:** ^1^ Booth School of Business University of Chicago Chicago Illinois USA; ^2^ Center for Healthcare Delivery Science and Innovation University of Chicago Chicago Illinois USA; ^3^ Department of Neurology, Biological Sciences Division University of Chicago Chicago Illinois USA; ^4^ Department of Medicine, Biological Sciences Division University of Chicago Chicago Illinois USA

Tweet 1: Empowering the front lines: how one hospital innovation center helped address the healthcare delivery challenges resulting from COVID‐19. #COVID19 #JHMChat #uchicago

Tweet 2: How can hospital innovation centers support healthcare value in times of crisis? #COVID19 #JHMChat #uchicago

## INTRODUCTION

As Medicare insolvency looms and healthcare financing moves toward value‐based payments, investment in healthcare delivery innovation has become critical to strengthening clinical outcomes while lowering costs (i.e., improving value).^1^, ^2^ In fact, healthcare innovation is often defined as “a new match between a need and a solution that creates better value than what currently exists.”^3^ In the United States, one of the signature investments in healthcare innovation is the Center for Medicare and Medicaid Innovation (CMMI) at the Centers for Medicare & Medicaid Services (CMS).^4^ But investments in healthcare delivery innovation are not limited to payors. Healthcare provider organizations have begun to support innovation centers as well.^5^, ^6^, ^7^

Although the aims of healthcare system innovation centers can be diverse, including a focus on technology development,^6^, ^7^ many are focused on real‐time healthcare delivery problem solving,^5^, ^7^ and employ a variety of methods to address identified challenges, including contextual inquiry to better define problems; crowdsourcing to inform solutions; and rapid cycle mini‐pilots for evaluation.^8^ Centers have also invested in new skillsets to support activities, including human‐centered design and computer programming.^5^, ^9^ Generalists (including hospitalists) head many of these centers, owing to their frequent leadership of quality, informatics, and value initiatives across healthcare systems nationally.

Given the aims, approaches, and skillsets of healthcare system innovation centers, they are well‐positioned to provide value in the context of rapidly emerging threats to healthcare delivery, such as the COVID‐19 pandemic, where they can empower the healthcare workforce, including those on the front lines, to share and implement potential solutions they have identified. Here, we provide an example of how one innovation center used an innovation tournament to rapidly address the healthcare delivery uncertainties resulting from the COVID‐19 pandemic, resulting in higher value care.

## CASE STUDY

### Setting

The Center for Healthcare Delivery Science and Innovation was established at the University of Chicago Medical Center (UCMC) in 2016 through a generous donor contribution with matching institutional support. The Center's mission is “to connect scholars and leaders across UCMC and beyond to catalyze innovation and discovery in healthcare delivery,” achieving this mission by providing grants, educational opportunities, logistical support for healthcare delivery research, and nourishing a community of scholars and leaders interested in healthcare delivery science and innovation. The Center supports three masters‐level full‐time staff, including an administrative director, project manager, and coordinator, as well as 0.25 full time equivalent (FTE) of a staff data scientist and 0.2 FTE of faculty in data and implementation science. The Center's physician director is UCMC's Chief Quality and Innovation Officer.

### Innovation tournament

On March 31, 2020, the Innovation Center used broad and targeted email communications to notify UCMC's staff, faculty, and trainees of a COVID‐19 “Innovation Challenge”—an opportunity to “submit an idea that can be forwarded to administrative and clinical leaders, or request support to design, implement, and/or evaluate innovative solutions to address the unique COVID‐19 healthcare delivery challenges before us.” Multidisciplinary collaboration was encouraged. The Center committed to reviewing ideas and requests for support on a rolling basis and responding within 5–7 business days. Proposals were submitted electronically through the Center website (Appendix A). Support to review and respond to submissions included two administrative FTEs and 0.25 FTE of the Physician Director. For select proposals, the Center provided resources such as pilot funding (up to $10,000/proposal), design expertise, project management, biostatistical support, or facilitated connections to university or community resources.

### Descriptive analysis

In the first 10 weeks (March 31 to June 10, 2020), the Center received 182 proposals, with most (58%) submitted in the first week. Proposals were categorized by theme, most commonly addressing: personal protective equipment (PPE) for healthcare workers and/or the community (27%, *n* = 50); awareness by employees or the community of COVID‐19 information (18%, *n* = 32); and the design of devices and supplies to prevent COVID‐19 (12%, *n* = 21). Other themes addressed testing, mobile application development, and data analytics, among others. Staff (43%, *n* = 79), faculty (27%, *n* = 50), and housestaff (13%, *n* = 23) submitted proposals, particularly those in the Department of Medicine (16%, *n* = 29) and Nursing (11%, *n* = 20).

Overall, 74 (41%) proposals were considered for resource support other than grant funding, 14 (8%) were considered specifically for grants, and 50 (28%) were suggestions forwarded to clinical or administrative leaders. Thirty‐one (17%) proposed projects “already in progress” and 13 (7%) were out of scope. Approximately $35,000 in funding was provided to eight proposals. Four received biostatistical support, and project management and design services were provided to additional proposals. Proposal review and decisions occurred in a mean of 8.6 (±5.2) business days.

### Examples of supported proposals

One faculty member submitted a proposal to address the community's social needs by making available at no cost for 6 months a proprietary electronic tool, originally developed by her research team, to match patients' needs to relevant community resources.^10^ The Center provided project management, including identifying end‐users, establishing tool access and training, and evaluating use. In the first 4 months (May to September 2020), approximately 400 unique users (most commonly from Social Work, Population Health, and Community Health) made approximately 2500 social service referrals (most often for food, housing, utility assistance, counseling, transportation, and/or childcare). The pilot resulted in a 3‐year contract with the health system.

Another faculty requested support to create a graphic narrative to teach youth in under‐resourced communities about COVID‐19 prevention. This resulted in the Center funding the development of an animated video titled “One Day at a Time,” which was launched July 21, 2020, and marketed widely, with approximately 5000 views to date.^11^

A cardiology fellow requested support to develop a mobile application to provide employees with quick access to hospital COVID‐19 policies, as well as all inpatient room phone numbers to facilitate remote patient rounding. As a result of the Center's funding and project management, over 1000 unique users downloaded the app within 4 months of release, with policy content views peaking at 801 weekly views, and patient room phone number dialing peaking at 277 weekly calls.^12^

The Center received multiple related submissions from staff to support the appropriate use of PPE, resulting in collaboration with a design firm to develop new hospital PPE signage. The Center's project management and funding resulted in the creation of fifteen 8 foot banners and 2400 laminated signs distributed across the medical center. Designs shared on the internet beginning April 12, 2020, had over 2000 views in the first week.^13^ As part of a multimodal approach, this work resulted in staff knowledge of appropriate PPE practices greater than similar staff globally.^14^

Additional examples of supported proposals, including supported research studies, are described in Table 1.

## DISCUSSION

Results suggest that the tournament was an effective approach to engage the healthcare workforce during a crisis and generate innovation from within the healthcare system, in a relatively short period of time and for a relatively low budget. The Center received proposals from a broad range of applicants and was able to respond in a timely manner, including forwarding proposals to appropriate healthcare system leaders (Table 1).

**Table 1:** Five other examples of proposals supported through the COVID innovation challenge

Submission description	Support provided and results to date
Request from emergency medicine faculty to develop inexpensive ($100–200) electricity‐free ventilator that could be used in resource constrained settings globally.	A prototype^15^ based on concepts used to design an electricity‐free neonatal ventilator^16^, ^17^ was produced. Funding was provided for preclinical validation to demonstrate the device's reliable functionality at pressures required for mechanically ventilating an adult using the university's test lung simulator. This included funds for supplies for prototype iteration and testing. The Center also connected the team to expertise from the University's Technology Commercialization Center and a local incubator focused on device development. The device was shown to deliver stable pressures and control of respiratory rate comparable to a standard critical care ventilator, with a publication currently under peer review.
Request from infectious diseases faculty to support retrospective analysis of the first cohort of COVID‐19 patients admitted to our institution.	Provided funding and biostatistical support. The analysis described the cohort's clinical presentation, demographics, comorbidities, hospital course, disposition, and mortality, and was subsequently published.^18^
Request from director of physician relations to help identify faculty who could provide COVID‐19 practice updates relevant to community providers.	The Center identified relevant topics and lecturers. Four virtual lectures were given in May and June 2020. Lectures addressed telemedicine, clinical pathways, PPE and compassion fatigue, and were given by experts in informatics, infection control, and psychiatry, respectively. One hundred and forty‐six community providers attended lectures, including numerous international attendees from Mexico and South America.
Request from junior surgical faculty to support development of online calculator to facilitate completion of a scoring system to identify MeNTS^19^ procedures in the context of COVID‐19.	Provided connections and project management to facilitate visualization of the MeNTS score in the electronic health record and create an online calculator using REDCap.^20^
Request from infectious diseases faculty for support to determine clinical characteristics associated with false‐negative SARS‐CoV‐2 test results to help inform COVID‐19 testing practices in the inpatient setting.	Provided funding and biostatistical support. Of the initial 1009 SARS‐CoV‐2 test results analyzed, 4% were false‐negatives.^21^ Using multivariable regression, false‐negative test results were strongly and significantly associated with anosmia/ageusia, COVID‐19+ contacts, and elevated lactate dehydrogenase levels on hospital presentation.

Abbreviations: COVID‐19, coronavirus disease 2019; MeNTS, Medically‐Necessary Time‐Sensitive; PPE, personal protective equipment; REDCap, Research Electronic Data Capture; SARS‐CoV‐2, severe acute respiratory coronavirus virus 2.

Several healthcare systems including our own^22^ have previously used innovation tournaments to crowdsource solutions and encourage front line engagement,^22^, ^23^, ^24^, ^25^ but this tournament was unique. Given the urgency of the challenges posed by the pandemic, the tournament was established rapidly, and the Center committed to responding to proposals quickly. This necessitated streamlining the proposal review process, precluding more extensive reviews by expert panels and judges, as described in prior tournaments.^25^ Despite this, we were unable to meet our goal response time, even with over two dedicated FTE. Defining scope was also a unique challenge, given the uncertainties surrounding a rapidly evolving crisis involving an unknown pathogen. Because of this, we chose to conceptualize healthcare delivery innovation broadly, rather than simply focusing on any one area such as digital tools; despite this broad definition, we still struggled with whether a number of excellent proposals were in scope, ultimately choosing not to pursue many after categorizing them as *clinical trials* instead. For example, a faculty member requested funds for supplies to measure the cytokine storm experienced by critical care patients, to help target anti‐inflammatory therapy to those in need. In lieu of supporting such requests, we referred them to another funding opportunity from our university's clinical trials center.

Our analysis has limitations. First, we did not have the resources to track the results of all connections made for those proposing initiatives already in progress. However, many applicants suggested they leveraged these connections. Examples include (1) an idea from a trainee to decontaminate N95 masks for reuse using an established protocol,^26^ which we referred to the Supply Chain team already beginning such an initiative and (2) many proposals related to 3D printing of face shields and sewing of fabric face masks, which we referred to local initiatives in progress.

We also did not have the resources to track the results of all suggestions forwarded to healthcare system leaders, although we know at least some were implemented. For example, a nurse submitted an idea for utilizing intravenous pumps outside of patient rooms to minimize room entry to conserve PPE and reduce infection exposure, which was implemented across select hospital units. Regardless of whether suggestions were ultimately implemented, sharing them gave leadership the opportunity to receive input from the front line, while giving the front line an opportunity to provide input to leadership. Past work suggests that facilitating such information flow can promote employee engagement, in turn enhancing patient safety and reducing employee turnover, ultimately leading to higher value care.^24^, ^25^, ^27^

Despite our ability to describe the impact of our initiative on the development and use of innovative products, tools, and services, and the conduct and completion of impactful research studies, our description is limited in that it is unable to assess impact on clinical outcomes.

Our approach to crowdsourcing solutions from the front line to address healthcare crises is generalizable beyond the COVID‐19 pandemic. In addition, ideas generated from this tournament have informed other innovation initiatives in our healthcare system, such as the American Board of Internal Medicine (ABIM) Foundation's “Choosing Wisely” campaign, which we participate in annually.^22^

In conclusion, our experience suggests that, as part of an institutional response to a crisis, a healthcare system innovation center can efficiently empower the front line, support potential solutions, and facilitate referral of solutions to relevant entities within the organization. Such a response can be provided with modest staffing and funding, but requires specialized services (such as design and statistical services) and relationships with senior operational leaders. Future work should more closely examine the impact of innovation centers and tournaments on employee engagement and the value of care provided.

## CONFLICTS OF INTEREST

The authors declare no conflicts of interest.

## ETHICS STATEMENT

This project was formally determined to be Quality Improvement, not human subjects research, and was therefore not reviewed by the Institutional Review Board, which is consistent with institutional policy.
